# Inflammatory Co-Regulation of Voltage-Gated Sodium Channels and Na,K-ATPase in Metastatic Breast Cancer

**DOI:** 10.3390/ijms27010424

**Published:** 2025-12-31

**Authors:** Steven D. Scahill, Kelly Jean Sherman, Dennis Paul

**Affiliations:** 1 Department of Interdisciplinary Oncology, Louisiana State University Health Sciences Center, New Orleans, LA 70112, USA; 2 Department of Pharmacology and Experimental Therapeutics, Louisiana State University Health Sciences Center, New Orleans, LA 70112, USA; ksherm@lsuhsc.edu (K.J.S.); dpaul@lsuhsc.edu (D.P.)

**Keywords:** sodium transport, metastasis, VGSC, Na,K-ATPase, inflammation, breast cancer

## Abstract

Sodium regulation is a potentially major driver of cancer metastasis. Voltage-gated sodium channels (VGSCs) and Na,K-ATPase are sodium transporters that are upregulated in many advanced carcinomas and are implicated as metastatic drivers. However, little is known about what drives this overexpression, how these proteins influence metastatic behavior, or whether these complementary sodium transporters are co-regulated in cancer. Using sodium transporter regulation in healthy neurons as a model, the present study demonstrated that the inflammatory mediator tumor necrosis factor alpha (TNFα) affects the expression of VGSCs and Na,K-ATPase in an in vitro model of metastatic breast cancer. Acute TNFα challenge increased RNA for sodium transporter subtypes by 20–100%, TNFα reduced the overall expression of VGSCs by 20–30% at all time-points examined, and long-term administration increased nuclear localization of the α1 subtype of Na,K-ATPase while increasing the overall expression of the α3 subtype. This study established that VGSCs and Na,K-ATPase are co-regulated by TNFα at the RNA level, and it was demonstrated that both TNFα and sodium transport-blocking drugs can significantly impact cellular metastasis-like behavior. Together these data are evidence that inflammation in metastatic breast cancer co-regulates the expression of VGSCs and Na,K-ATPase, and this regulatory system may contribute to carcinogenesis.

## 1. Introduction

The vast majority of cancer deaths are attributable to metastatic cases of the disease [1]. Therefore, understanding pathways that drive metastasis in cancer is essential for the effective prevention of morbidity and mortality in cancer patients. In recent years, ion regulation has gained prominence as a potentially significant driver of tumorigenesis and metastasis [2]. Altered regulation of intracellular ions can lead to cell proliferation, acidification of the extracellular matrix, activation of signal transduction pathways, and altered gene expression in ways that can promote cancerous progression [2,3,4]. Sodium regulation, in particular, is an attractive target in cancer as tumorous tissue in patients has a measurably higher intracellular sodium content than healthy tissue [5,6]. Voltage-gated sodium channels (VGSCs) and Na,K-ATPase are two major sodium-transporting proteins that have been linked to cancerous progression in an increasing number of studies [7,8,9,10].

VGSCs are transmembrane proteins consisting of a pore-forming α subunit that typically associates with one or two β subunits [7]. There are nine α subtypes (Na_v_1.1-Na_v_1.9) that exhibit tissue-specific expression profiles in healthy cells. Classically, VGSCs are known to be robustly expressed on the plasma membranes of depolarizing cells like neurons and cardiomyocytes, where they open in response to changes in membrane potential, leading to a rapid depolarizing influx of sodium ions into the cell. VGSCs are also expressed at lower levels in non-depolarizing cells, where even in the absence of full action potentials they can facilitate persistent inward sodium currents and have been linked to both healthy and pathogenic function [11,12,13]. Crucially, VGSCs are overexpressed in a diverse array of cancers, particularly in metastatic carcinomas [7,8]. In addition to being a negative prognostic marker for patient outcomes, VGSC expression is reported to contribute actively to cancer cell invasiveness in vitro and metastasis in vivo [3,8,14,15]. However, little is known about how these sodium channels become upregulated in cancer cells or how they drive metastatic development.

Na,K-ATPase, also known as sodium pumps, are energy-dependent ion transporters that use ATP to power the transport of three sodium ions out of a cell in an antiport fashion, with two potassium ions passing into a cell against their concentration gradients [16,17]. They consist of a catalytic α subunit, a β subunit that facilitates intracellular transport, and a γ subunit that modulates pump activity [16,17]. Interestingly, Na,K-ATPase acts not only as an ion transporter but as a receptor. It binds to endogenous ouabain-like compounds in the body to trigger signal transduction pathways, including the Src kinase pathway, the MAPK pathway, and the PI3K-Akt pathway [16,18]. The Na,K-ATPase α1 subtype is expressed fairly ubiquitously in human cells, while the α2 and α3 subtypes are expressed in a more tissue-specific pattern [17]. Like VGSCs, Na,K-ATPase are often over-expressed in cancer cells and have also been correlated specifically with metastasis [9,10]. Also, as with VGSCs, there is little known about the upstream regulation of Na,K-ATPase in cancer or how it contributes to metastatic progression.

Although there is a growing body of research into the contributions of VGSCs or Na,K-ATPase individually in a variety of cancer models, they have not been considered as a cooperative or co-regulated unit. This is despite the fact that they have complementary activity on sodium ions, overlaps in their reported downstream effectors, like ERK1/2 or EGFr signaling, and that they are both upregulated in a variety of metastatic cancers [14,16,19,20,21]. If indeed these sodium transporters are co-regulated in cancer cells, understanding this relationship would help elucidate the larger regulatory mechanism modulating these proteins in cancer and driving their subsequent impacts on metastatic progression. Effectively mapping the regulation of these sodium transporters in cancer could have significant clinical implications, as early studies examining the effects of VGSC or Na,K-ATPase-blocking drugs in cancer patients have, thus far, proved inconclusive [22,23,24,25].

While there is currently no definitive indication of how VGSCs and Na,K-ATPase are regulated independently or conjointly in cancer cells, neurons may provide a relevant mechanistic model, demonstrating how these proteins are co-regulated by microenvironmental stimuli in healthy depolarizing cells. In pain-sensing neurons, such as those in the Dorsal Root Ganglion (DRG), inflammatory mediators drive the overexpression of VGSCs on the cell membrane, which leads to increased rates of depolarization in the neurons, amplified pain signaling, and subsequent upregulation of Na,K-ATPase to repolarize the cells. Noting this relationship in healthy depolarizing cells, in the present study we tested the hypothesis that cancer cells may hijack this normal mechanism, responding to inflammatory signals in the tumor microenvironment to drive the overexpression of sodium transporters in cancer cells, in turn driving metastatic development. Beyond the neuronal model of pain sensitization, inflammatory signaling is a conspicuous candidate to drive metastatic processes in cancer cells. Inflammatory signals are ubiquitous throughout the tumor microenvironment, both from within the tumor as damaged cells release inflammatory mediators like tumor necrosis factor (TNF), and from outside of the tumor as invading immune cells exchange complex reciprocal signaling crosstalk with the cancer cells [26,27,28,29]. Inflammatory signals have also been shown to drive cancerous progression in both acute in vitro models, with inflammatory challenge promoting aggressive behavior in cell culture, and in vivo chronic inflammatory models with conditions like obesity or infectious disease serving as negative prognostic markers in patients [30,31,32,33,34,35].

With this study, we demonstrate the effects of TNFα on the expression of clinically relevant subunits of VGSCs and Na,K-ATPase at the RNA, protein localization, and protein expression levels in human metastatic breast cancer MDA-MB-231 cells. These cells are of particular relevance to this study as they are well known to robustly express the VGSC subtype Na_v_1.5, which directly contributes to the cells’ invasive potential [36]. Furthermore, these channels are known to affect the cells’ sodium homeostasis under normal physiological conditions, as electrophysiological studies have demonstrated that MDA-MB-231 cells display an inducible fast inward sodium current, which is partially activated at the cells’ resting potential [37]. Co-regulation of these two sodium transporters was examined at the RNA level, and the effects of TNFα and sodium transport-blocking drugs on the cells’ metastasis-like behavior was evaluated.

## 2. Results

### 2.1. Inflammatory RNA Regulation of Sodium Transporters After siRNA Knockdown

**Figure 1:**
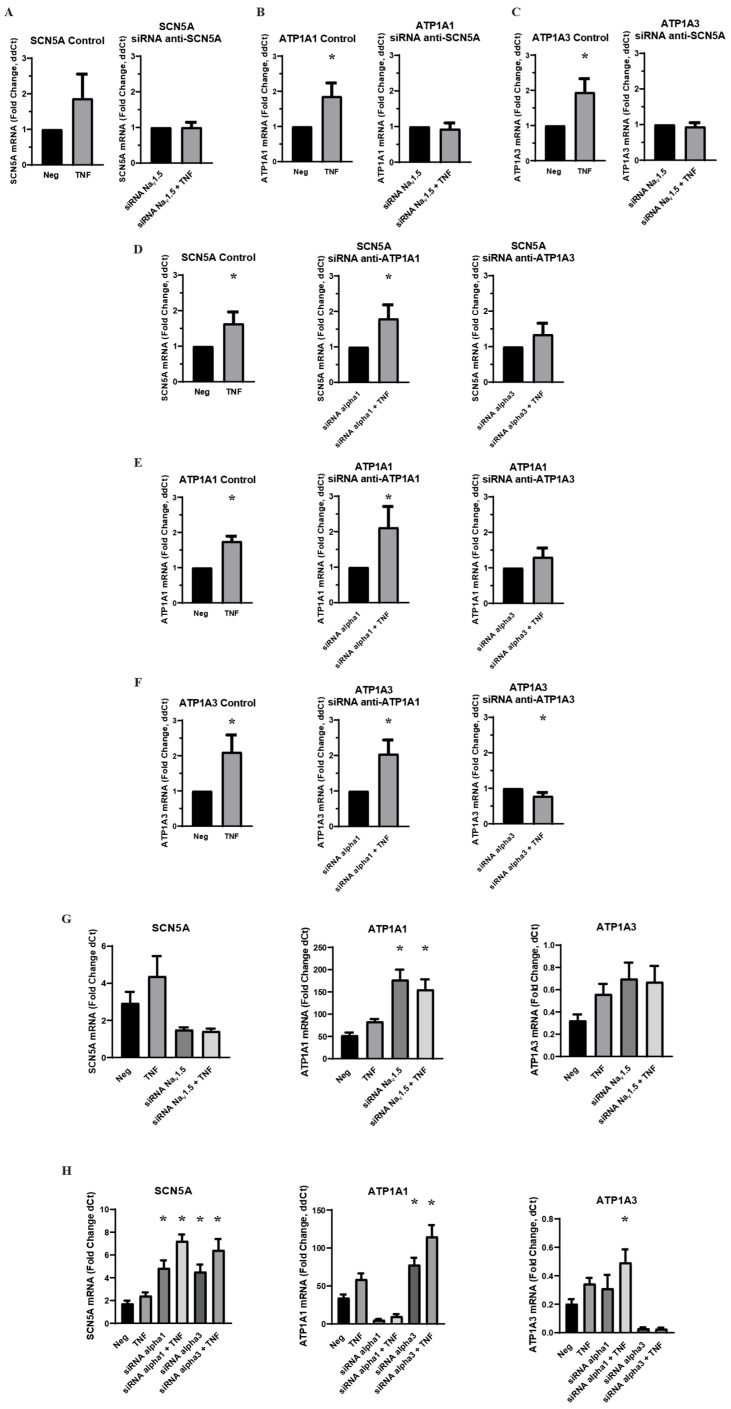
Effect of VGSC and Na,K-ATPase siRNA knockdown on inflammatory-mediated effect on target RNA expression in MDA-MB-231 cells (n = 8). (**A**) Effect of anti-*SCN5A* siRNA knockdown on *SCN5A* RNA expression. (**B**) Effect of anti-*SCN5A* siRNA knockdown on *ATP1A1* RNA expression. (**C**) Effect of anti-*SCN5A* siRNA knockdown on *ATP1A3* RNA expression. (**D**) Effect of anti-*ATP1A1* and anti-*ATP1A3* siRNA knockdown on *SCN5A* RNA expression. (**E**) Effect of anti-*ATP1A1* and anti-*ATP1A3* siRNA knockdown on *ATP1A1* RNA expression. (**F**) Effect of anti-*ATP1A1* and anti-*ATP1A3* siRNA knockdown on *ATP1A3* RNA expression. A-F RNA expression presented as fold-change of ΔΔCt values (2^−ΔΔCt^. ΔΔCt = ΔCt TNFα-treated sample − ΔCt paired negative control sample). Statistical significance of the differences between each experimental group RNA expression compared to paired control RNA expression was assessed via one-tailed *t*-test (* *p* < 0.05). (**G**) RNA expression presented as fold change of ΔCt values (2^−ΔCt^, ΔCt = target gene Ct − RER1 Ct) in control cells and anti-*SCN5A* siRNA transfected cells under negative treatment and TNFα treatment conditions. (**H**) RNA expression presented as a fold-change of ΔCt values (2^−ΔCt^, ΔCt = target gene Ct − RER1 Ct) in control cells and anti-*ATP1A1* and *ATP1A3* siRNA transfected cells under negative treatment and TNFα treatment groups. Statistical significance of the differences between experimental group ΔCt values and negative control ΔCt values was assessed via one-way ANOVA with a post-hoc Dunnett’s multiple comparisons test (* *p* < 0.05).

An acute challenge of a physiological concentration of TNFα was administered to MDA-MB-231 cells, and RT-qPCR was used to measure the effect on VGSC and Na,K-ATPase RNA. The sodium transporter subtypes examined were the VGSCs subtype Na_v_1.5, which is known to be upregulated in clinical breast cancer cases and in MDA-MB-231 cells, Na,K-ATPase α1, a ubiquitously expressed subtype of sodium pumps in human cells, and Na,K-ATPase α3, a subtype typically found in neurons among other specialized cells. RNA expression was evaluated for *SCN5A*, the gene coding for Na_v_1.5, *ATP1A1*, the gene coding for the α1 subtype of Na,K-ATPase, and *ATP1A3*, the gene coding for the α3 subtype of Na,K-ATPase. It was found that an acute 1 h application of TNFα induced a significant increase in RNA for all three transporter subtypes (Figure 1). This is evidence that inflammation can indeed regulate sodium channels and sodium pumps in MDA-MB-231 cells, either in a co-regulated or independent fashion. However, when MDA-MB-231 cells were transfected with siRNA against Na_v_1.5 prior to TNFα challenge, there was no longer any inflammatory increase in RNA for either the *ATP1A1* or *ATP1A3* subtypes of sodium pumps (Figure 1A–C). Conversely, when the cells were transfected with siRNA against Na,K-ATPase α1 or α3, there was a very similar inflammatory effect on *SCN5A* RNA, although this effect did not reach statistical significance in the α3 knockdowns (Figure 1D). These data are evidence of the inflammatory co-regulation of VGSCs and Na,K-ATPase in metastatic breast cancer cells in a manner analogous to pain-sensing neurons, with the inflammatory effect on VGSCs leading to the inflammatory effect on sodium pumps and not vice versa. However, the data indicates a more complex co-regulatory relationship between sodium channels and sodium pumps, as siRNA knockdown of Na_v_1.5 induced a basal increase in RNA for both Na,K-ATPase subtypes, and siRNA knockdown of Na,K-ATPase subtypes α1 or α3 induced a basal increase in VGSC RNA (Figure 1G,H).

### 2.2. Sodium Dependence of Inflammatory Regulation of VGSC and Na,K-ATPase RNA

**Figure 2:**
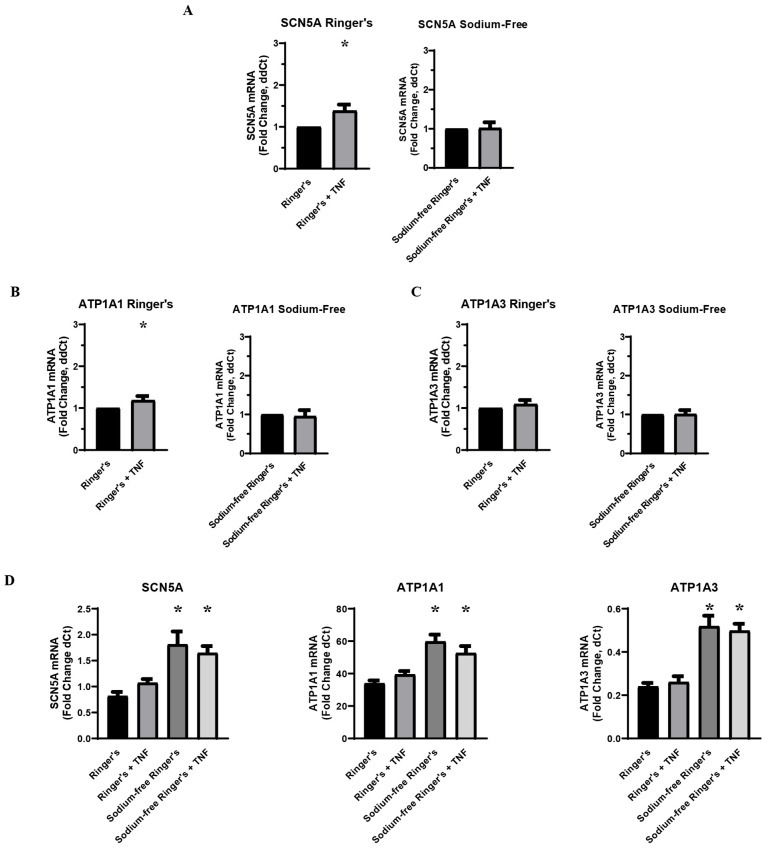
Effect of environmental sodium on inflammatory-mediated effect on VGSCs and Na,K-ATPase (n = 8). (**A**) TNFα-mediated effect on *SCN5A* RNA expression in a serum-free Ringer’s buffer with and without sodium. (**B**) TNFα-mediated effect on *ATP1A1* RNA expression in a serum-free Ringer’s buffer with and without sodium. (**C**) TNFα-mediated effect on *ATP1A3* RNA expression in a serum-free Ringer’s buffer with and without sodium. All data is expressed at a fold-change of ΔΔCt normalized to the negative control (2^−ΔΔCt^. ΔΔCt = ΔCt TNFα-treated sample − ΔCt paired negative control sample). Statistical significance of the differences between each experimental group RNA expression compared to paired control group RNA expression was assessed via one-tailed *t*-test (* *p* < 0.05). (**D**) RNA expression of *SCN5A*, *ATP1A1*, and *ATP1A3* presented as a fold-change of ΔCt values (2^−ΔCt^, ΔCt = target gene Ct − RER1 Ct) in Ringer’s buffer and sodium-free Ringer’s buffer. Statistical significance for the differences between experimental group ΔCt values and negative control ΔCt values was assessed via one-way ANOVA with a post-hoc Dunnett’s multiple comparisons test (* *p* < 0.05).

In order to determine the role of sodium ions in the inflammatory regulation of VGSCs and Na,K-ATPase, MDA-MB-231 cells were pre-incubated in a sodium-free Ringer’s buffer prior to acute challenge with TNFα. Because all other inflammatory challenge experiments were conducted with a base medium of DMEM with 10% FBS, which differs significantly in its constitution from Ringer’s buffer apart from sodium concentration, concurrent challenge experiments were performed with cells pre-incubated in Ringer’s Buffer with sodium included. RT-qPCR showed no change in RNA expression of *SCN5A*, *ATP1A1*, or *ATP1A3* following challenge with TNFα under sodium-free conditions, indicating that sodium is essential to the inflammatory regulation of these ion transporters (Figure 2A–C). In the cells that had been pre-incubated in Ringer’s buffer with sodium, the increase in *SCN5A* RNA in the TNFα-treated samples was in line with the expected approximate 20–60% increase that was observed in the previous inflammatory challenge experiments (Figure 2A). However, the increase in *ATP1A1* RNA in the TNFα-treated samples was well below the 50–100% increase observed in previous experiments, and there was no observed upregulation in *ATP1A3* RNA (Figure 2B,C). These data indicate that sodium is sufficient to recover the inflammatory regulation of VGSCs in metastatic breast cancer cells, while sodium is not in itself sufficient to recover in the inflammatory regulation of Na,K-ATPase. This is further evidence that sodium pumps are further down the regulatory pathway than sodium channels in response to inflammatory stress. It should also be noted that RNA for all three ion transporters was increased in the absence of sodium (Figure 2D). This demonstrates that intracellular sodium concentration is a regulatory factor in the transcriptional regulation of these proteins, which may account for the basal increase in Na,K-ATPase RNA observed following the knockdown of Na_v_1.5 (Figure 1G).

### 2.3. Effect of Pharmacological Blockade of VGSCs and Na,K-ATPase on Inflammatory RNA Regulation

**Figure 3:**
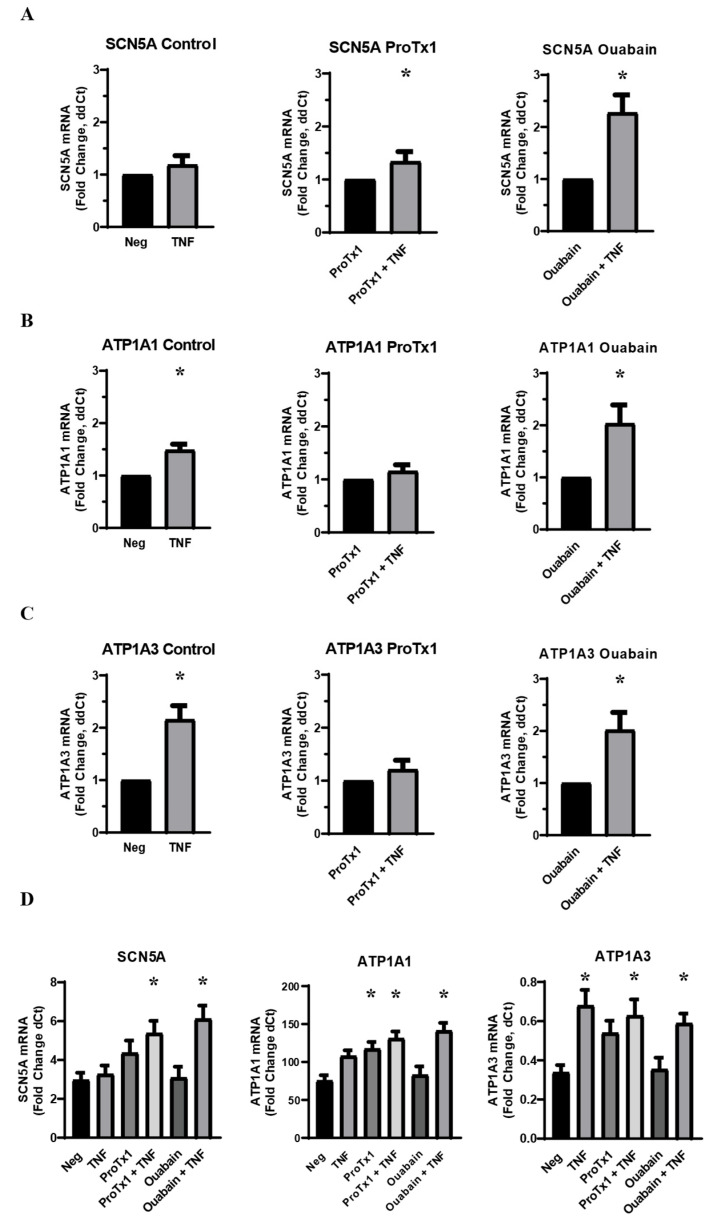
Effect of co-administration of TNFα with a sodium channel blocking drug (ProTx1) and sodium pump blocking drug (ouabain) on VGSC and Na,K-ATPase RNA expression in MDA-MB-231 cells (n = 8). (**A**) Effect of TNFα, ProTx1, and ouabain on *SCN5A* RNA expression. (**B**) Effect of TNFα, ProTx1, and ouabain on *ATP1A1* RNA expression. Data is expressed as a fold-change of ΔΔCt. (**C**) Effect of TNFα, ProTx1, and ouabain on *ATP1A3* RNA expression. Data in A–C is expressed as a fold-change of ΔΔCt (2^−ΔΔCt^. ΔΔCt = ΔCt inflammatory-treated sample − ΔCt paired negative control sample). Statistical significance of the differences between TNFα treatment group RNA expression compared to paired non-TNFα treatment control RNA expression for ΔΔCt was assessed via one-tailed *t*-test (* *p* < 0.05). (**D**) Effect of TNFα, ProTx1, and ouabain on *SCN5A*, *ATP1A1*, and *ATP1A3* RNA expression. Data is expressed as a fold-change of ΔCt (2^−ΔCt^, ΔCt = target gene Ct − RER1 Ct). Statistical significance for the differences between experimental group ΔCt values and negative control ΔCt values was assessed via one-way ANOVA with a post-hoc Dunnett’s multiple comparisons test (* *p* < 0.05).

In order to further investigate the role that sodium transport plays in the inflammatory co-regulation of VGSC and Na,K-ATPase RNA, MDA-MB-231 cells were again given an acute challenge of TNFα in a base medium of DMEM supplemented with 10% FBS. However, the TNFα was co-administered either with protoxin-1 (ProTx1), a sodium channel-blocking drug, or ouabain, which inhibits ion transport through Na,K-ATPase. RT-qPCR of RNA samples treated with TNFα and ProTx1 revealed that the VGSC blocker inhibited the inflammatory-mediated upregulation of both *ATP1A1* and *ATP1A3* RNA (Figure 3B,C). This indicates that normal ion transport though VGSCs is necessary for the inflammatory-mediated upregulation of Na,K-ATPase RNA. Conversely, when the TNFα was co-administered with ouabain, the inflammatory-mediated increase in *SCN5A* was not inhibited but was instead potentiated (Figure 3A). This effect may either be the result of the inhibited ion transport through the sodium pumps or the result of signal transduction pathways triggered by the binding of ouabain to Na,K-ATPase, as these represent the dual effects of the drug. Because this potentiation was not observed in the Na,K-ATPase siRNA knockdowns, the latter possibility seems most likely. In any case, the modulation of the inflammatory-mediated upregulation of *SCN5A* RNA by ouabain is further evidence of the unexpected complexity of the inflammatory co-regulation of VGSCs and Na,K-ATPase. Additionally, just as siRNA knockdown of Na_v_1.5 increased basal RNA for Na,K-ATPase RNA, so too did ProTx1 administration increase basal levels of RNA for both *ATP1A1* and *ATP1A3* (Figure 3D). Together these results indicate that the normal expression and ion-transporting function of VGSCs can impact the expression of Na,K-ATPase in either the presence or absence of inflammatory stress.

### 2.4. TNFα-Mediated Effects on Localization of VGSCs and Na,K-ATPase

**Figure 4:**
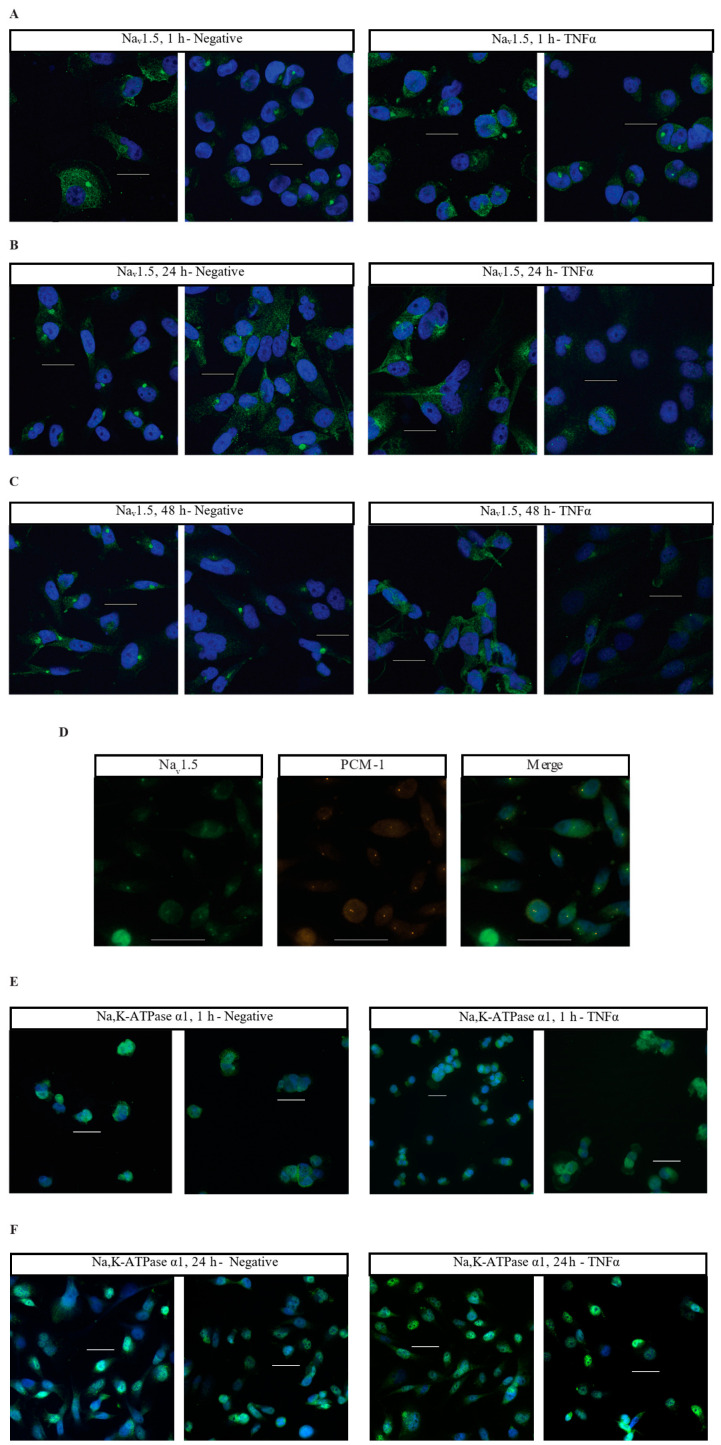

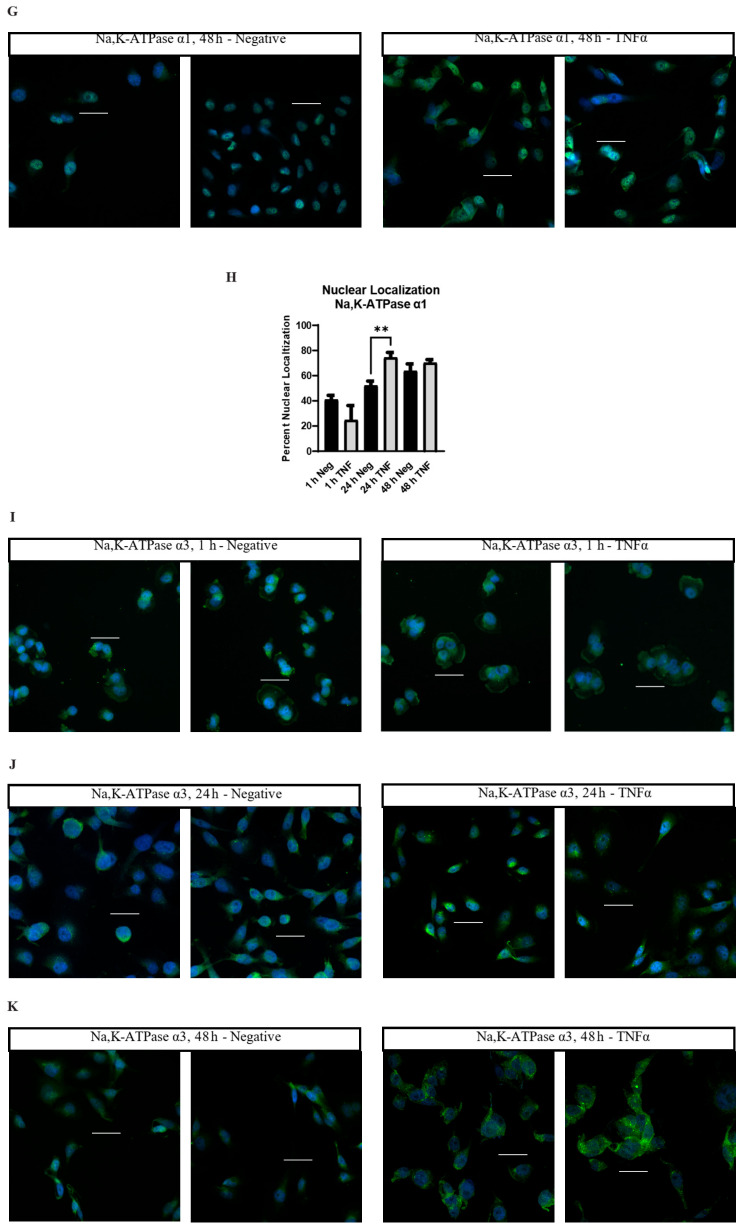
Immunocytofluorescent images of Na_v_1.5, Na,K-ATPase α1, and Na,K-ATPase α3 localization in MDA-MB-231 cells. (**A**) Cells stained with an anti-Na_v_1.5 antibody (green) and a DRAQ5 nuclear counterstain (blue). Cells were treated in normal cell culture medium with no additives (Negative) or 14.14 ng/mL TNFα. Cells were fixed and stained at 1 h post-treatment. (**B**) 24 h anti-Na_v_1.5 samples. (**C**) 48 h anti-Na_v_1.5 samples. (**D**) MDA-MB-231 cells co-stained with anti-Na_v_1.5 antibodies and antibodies targeting the centrosomal marker PCM-1. (**E**) Cells stained with anti-Na,K-ATPase α1 antibody (green) and DAPI nuclear counterstain (blue). Cells were fixed and stained at 1 h post-treatment. (**F**) 24 h anti-Na,K-ATPase α1 samples. (**G**) 48 h anti-Na,K-ATPase α1 samples. (**H**) The number of cells displaying nuclear localization of Na,K-ATPase α1 was counted and graphed as a percent of the total number of cells in each image taken. Statistical significance was assessed between negative and TNFα-treated samples at each time-point via two-tailed *t*-test (** *p* < 0.05). (**I**) Cells stained with anti-Na,K-ATPase α3 antibody (green) and DAPI nuclear counterstain (blue). Cells were fixed and stained at 1 h post-treatment. (**J**) 24 h anti-Na,K-ATPase α3 samples. (**K**) 48 h anti-Na,K-ATPase α3 samples. White scale bar = 25 µm.

Having characterized the acute inflammatory co-regulation of VGSCs and Na,K-ATPase at the RNA level, immunocytofluorescence was utilized to examine how TNFα alters the localization of VGSCs and Na,K-ATPase over time. MDA-MB-231 cells were treated in either basal cell culture medium or medium with added TNFα and incubated for 1 h, 24 h, or 48 h. At each terminal time-point, cells were fixed and stained with antibodies against Na_v_1.5, Na,K-ATPase α1, or Na,K-ATPase α3 with a nuclear counterstain. At 1 h, there was no evident difference in Na_v_1.5 localization between negative and TNFα-treated cells, with staining evident in the cytosol along some cell membranes, and with distinct punctate staining in the perinuclear region (Figure 4A). At 24 and 48 h, the control cells maintained patchy cytosolic staining with frequent perinuclear punctae for Na_v_1.5. However, at these later time-points, the staining for Na_v_1.5 in the TNFα-treated cells became more diffuse with far less frequent perinuclear punctae (Figure 4B,C). This is evidence that chronic TNFα administration alters the localization of Na_v_1.5 in MDA-MB-231 cells. The particular punctate staining pattern that was lost in the TNFα-treated cells appeared to match the expected localization of the cells’ centrosomes. This centrosomal co-localization of Na_v_1.5 was confirmed by co-staining with the centrosome marker PCM-1 (Figure 4D).

Na,K-ATPase α1 displays a heterogenous staining profile in MDA-MB-231 cells. A certain population of cells displays staining in the cytosol and cell membrane while being excluded from the nucleus. A second population displays significant nuclear co-localization of Na,K-ATPase α1 (Figure 4E–H). At 1 h, the minor population of cells demonstrates nuclear co-localization of Na,K-ATPase α1 in both control (40%, 66/165 cells) and TNFα-treated cells (28.2%, 33/117 cells) (Figure 4E). At 24 h, the proportion of cells displaying nuclear co-localization of Na,K-ATPase α1 increases under both conditions, but the proportion is noticeably higher in the TNFα-treated cells, with approximately 75.8% of cells (175/231 cells) in the TNFα group and 52.6% of cells (122/232 cells) in the control group displaying apparent nuclear co-localization (Figure 4F). This trend continued at 48 h, albeit with less of a difference between groups, with approximately 69.7% of cells (221/317 cells) in the TNFα group and 62.5% of cells (170/272 cells) in the control group displaying apparent nuclear co-localization (Figure 4G). Graphing the proportion of apparent nuclear localization in all the imaged cells reveals that the nuclear localization of Na,K-ATPase α1 increases as the cells are maintained in culture, but that this process happens more quickly in TNFα-treated cells (Figure 4H). This is evidence that TNFα increases the nuclear localization of Na,K-ATPase α1 in MDA-MB-231 cells.

Unlike Na_v_1.5 and Na,K-ATPase α1, TNFα does not appear to alter the localization of Na,K-ATPase α3, with MDA-MB-231 cells displaying staining along the cell membrane and in the cytosol, with particularly prominent staining in the perinuclear region in many cells (Figure 4I–K). However, while the localization of the α3 subunit does not appear to be altered by TNFα, there is noticeably brighter staining in the perinuclear region in many of the treated cells at 24 and 48 h as compared to the controls (Figure 4J,K). This may indicate an effect of chronic TNFα administration on the overall expression of Na,K-ATPase α3.

### 2.5. TNFα-Mediated Effects on Overall Expression of VGSC and Na,K-ATPase Protein

**Figure 5:**
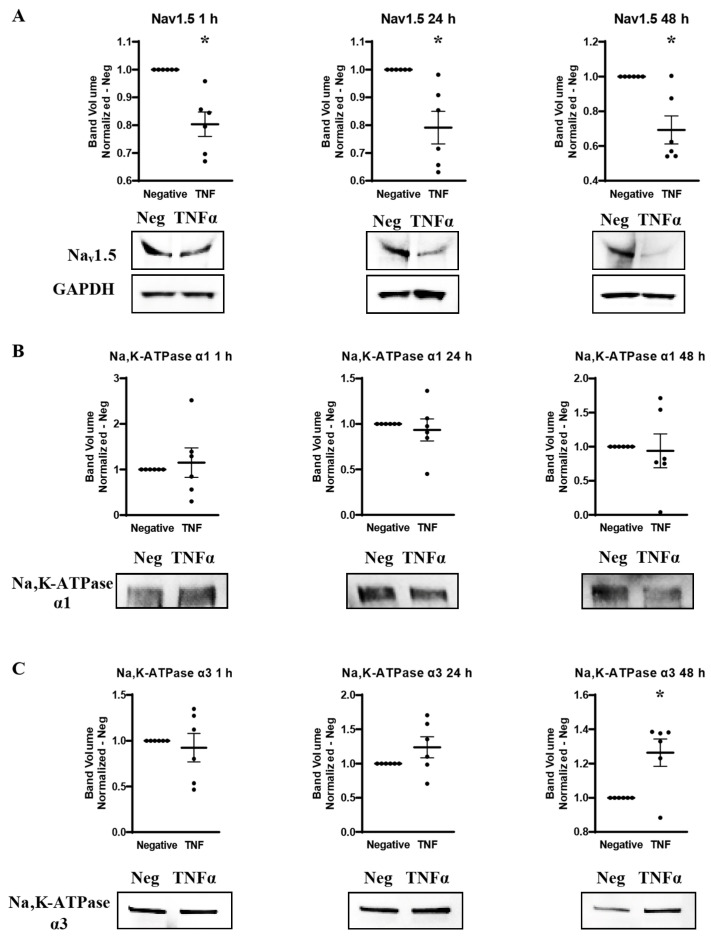
Overall protein expression of Na_v_1.5, Na,K-ATPase α1, and Na,K-ATPase α3 assessed via western blot in response to TNFα at 1 h, 24 h, and 48 h in MDA-MB-231 cells (n = 6). (**A**) Na_v_1.5 expression. (**B**) Na,K-ATPase α1 expression. (**C**) Na,K-ATPase α3 expression. Images representative of independent experiments. Data includes graph of protein expression (normalized TNFα-treated band volume relative to paired non-TNFα-treated control band volume). Statistical significance of the differences between TNFα-treated protein expression compared to paired non-TNFα-treated protein expression was assessed via two-tailed *t*-test (* *p* < 0.05).

Western blots were utilized to measure the effect of TNFα administration on the overall expression of Na_v_1.5, Na,K-ATPase α1 and Na,K-ATPase α3 protein over time. MDA-MB-231 cells were incubated in either basal cell culture medium or medium with added TNFα, and protein was extracted at 1 h, 24 h, and 48 h. Western blot was then performed with antibodies against Na_v_1.5, Na,K-ATPase α1, or Na,K-ATPase α3. Na,K-ATPase α1 and α3 proteins were normalized against total protein in the lane. Na_v_1.5, which required an adjusted protocol to resolve the protein bands, was normalized to GAPDH. It was found that TNFα significantly reduces the overall expression of Na_v_1.5 by approximately 20–30% at all time-points examined (Figure 5A). This may indicate that TNFα Na_v_1.5 induces internalization and proteasomal degradation, which is part of its normal regulatory cycle, at a rate that outpaces any de novo production, even at the 1 h time-point where *SCN5A* RNA was elevated [38]. Na,K-ATPase α1 showed no apparent alteration in overall protein expression at any time-point when challenged with TNFα (Figure 5B). However, long-term challenge with TNFα induced a trend towards increased expression of the Na,K-ATPase α3 subtype with a statistically significant increase in expression observed at 48 h (Figure 5C). This, along with the immunocytofluorescent studies, is evidence that chronic TNFα exposure may increase the overall expression of Na,K-ATPase α3 protein in MDA-MB-231 cells.

### 2.6. Effect of Pharmacological Blockade of VGSCs and Na,K-ATPase on Inflammatory Protein Regulation

**Figure 6:**
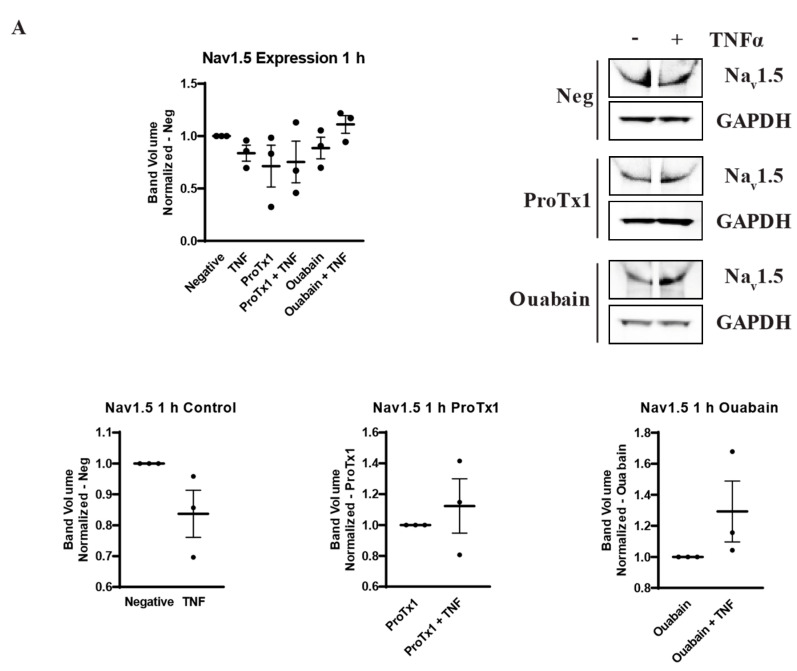

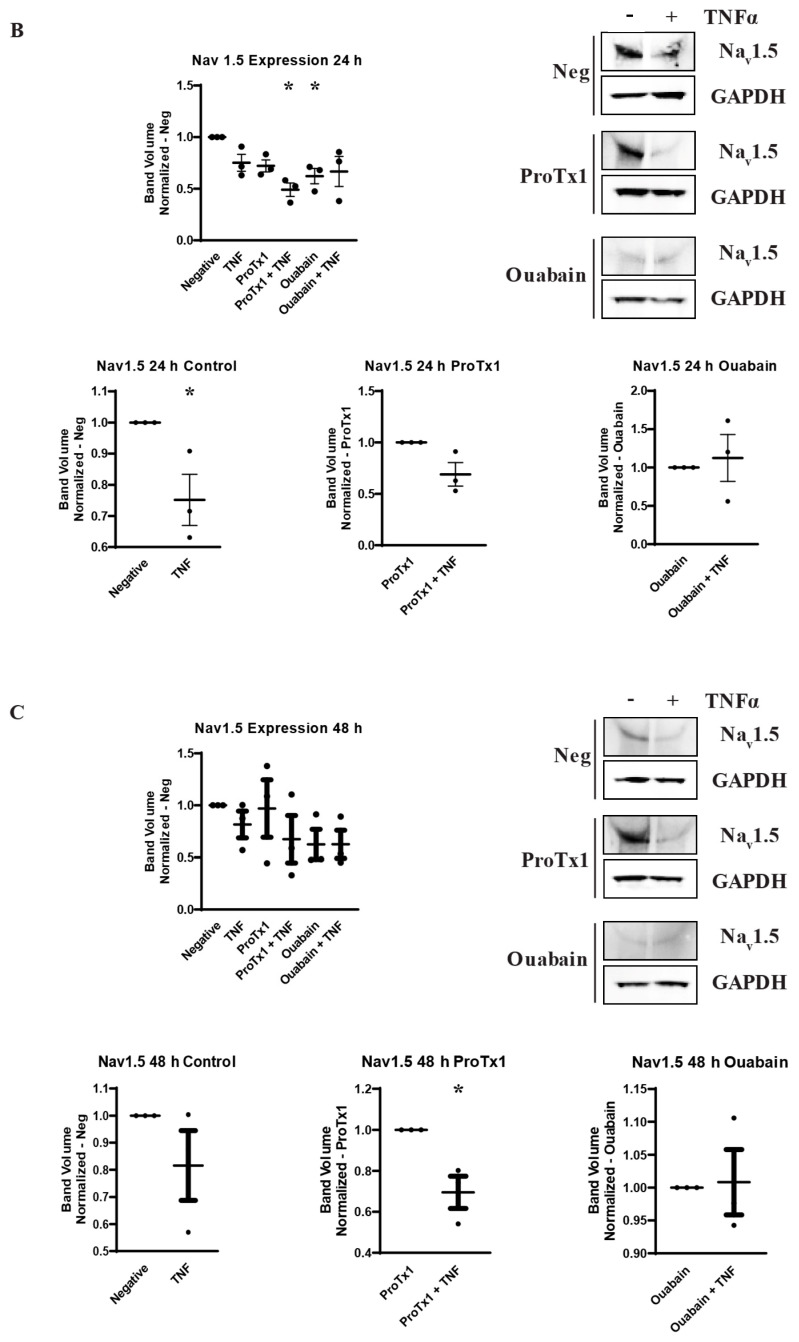

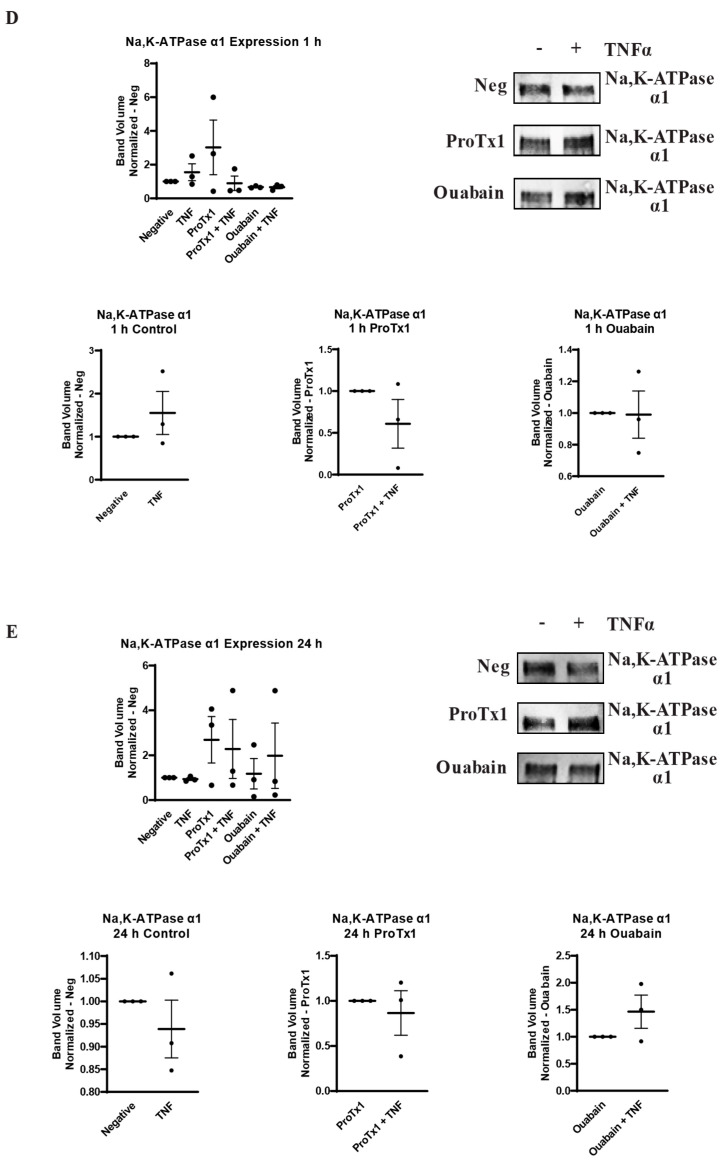

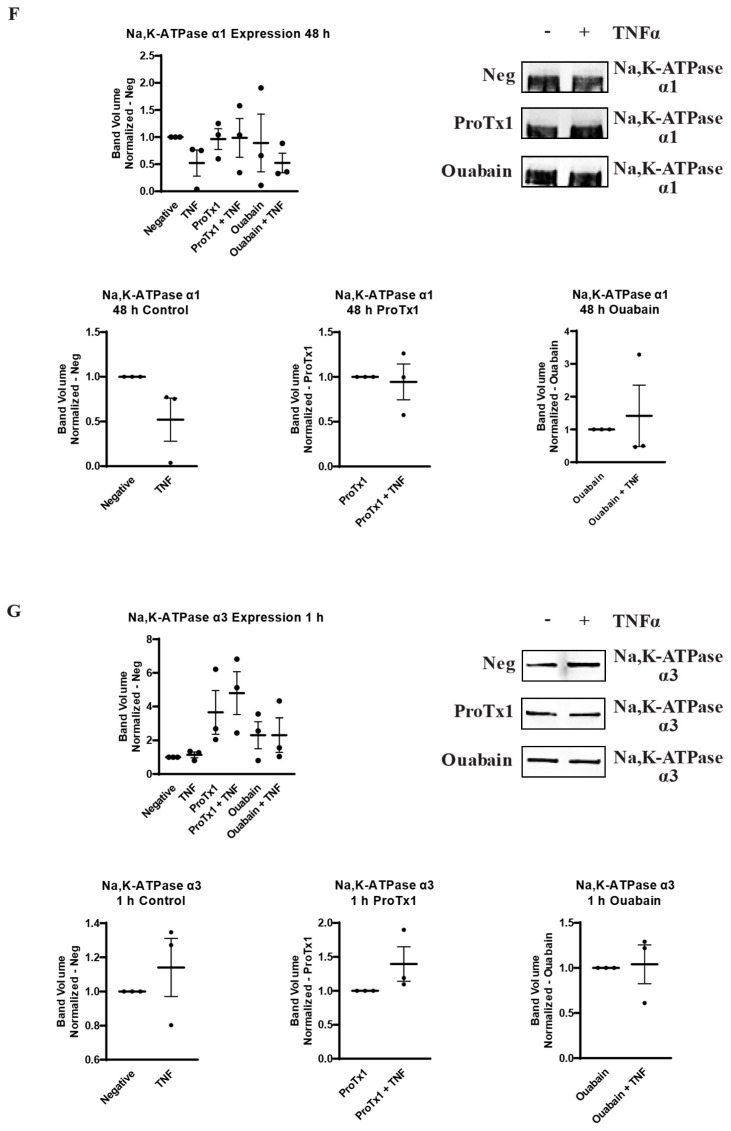

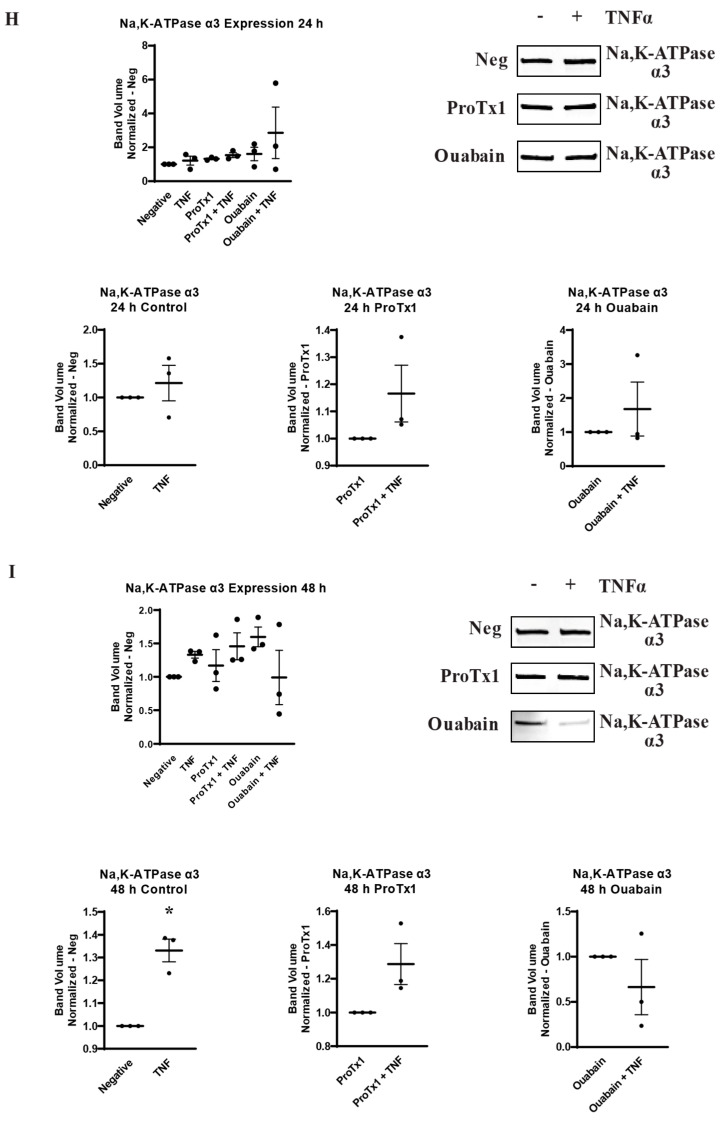
Overall protein expression of Na_v_1.5, Na,K-ATPase α1, and Na,K-ATPase α3 assessed via western blot in response to TNFα, ProTx1 and ouabain in MDA-MB-231 cells (n = 3). (**A**) Na_v_1.5 expression after 1 h. (**B**) Na_v_1.5 expression after 24 h. (**C**) Na_v_1.5 expression after 48 h. (**D**) Na,K-ATPase α1 expression after 1 h. (**E**) Na,K-ATPase α1 expression after 24 h. (**F**) Na,K-ATPase α1 expression after 48 h. (**G**) Na,K-ATPase α3 expression after 1 h. (**H**) Na,K-ATPase α3 expression after 24 h. (**I**) Na,K-ATPase α3 expression after 48 h. Images representative of three independent experiments. Data includes graph of protein expression in all groups (normalized experimental group band volume relative to paired normalized negative control band volume), and graphs of individual treatment groups (normalized TNFα-treated band volume relative to paired non-TNFα-treated control band volume). Statistical significance between each experimental group and negative controls was assessed via one-way ANOVA with a post-hoc Dunnett’s multiple comparisons test (* *p* < 0.05). Statistical significance of the differences between TNFα-treated protein expression compared to paired non-TNFα-treated protein expression was assessed via two-tailed *t*-test (* *p* < 0.05).

In order to explore how pharmacological blockade of VGSCs and Na,K-ATPase would affect the inflammatory regulation of the protein expression of these sodium transporters, western blot was performed on MDA-MB-231 cells that had been incubated in TNFα with the addition of the sodium channel blockers ProTx1 or the sodium pump blocker ouabain. Protein was extracted at 1 h, 24 h, and 48 h, and western blot was then performed with antibodies against Na_v_1.5, Na,K-ATPase α1, or Na,K-ATPase α3. Although these experiments were conducted with a limited sample size (n = 3), which prevents definitive conclusions from being reached, the results indicate possible complexities in the inflammatory regulation of VGSCs and Na,K-ATPase that should be explored in further experiments. At 1 h, co-administration of both ProTx1 and ouabain inhibited the TNFα-mediated downregulation of Na_v_1.5 (Figure 6A). Ouabain co-administration with TNFα in particular trended towards an increase in Na_v_1.5 in a manner reminiscent of the potentiation of the inflammatory upregulation of *SCN5A* at 1 h with ouabain and TNFα at the RNA level (Figure 3A). At 24 and 48 h, ProTx1 treatment appeared similar to control conditions, while long-term ouabain administration appears to downregulate the expression of Na_v_1.5 regardless of inflammatory challenge in a manner that is consistent with the effect of chronic ouabain administration on neurons (Figure 6B,C) [39]. Administration of ProTx1 or ouabain had no apparent effect on the basal or inflammatory-mediated expression of Na,K-ATPase α1 (Figure 6D–F). However, while both TNFα alone and TNFα co-administered with ProTx1 induced a trending increase in Na,K-ATPase α3 expression at 48 h, TNFα co-administered with ouabain induced a trending decrease in Na,K-ATPase α3 expression (Figure 6I). Together, these data provide evidence that both ProTx1 and ouabain can alter the inflammatory regulation of VGSC and Na,K-ATPase protein expression in ways that warrant further exploration.

### 2.7. Effect of TNFα, ProTx1 and Ouabain on Metastatic-like Behavior of MDA-MB-231 Cells

**Figure 7:**
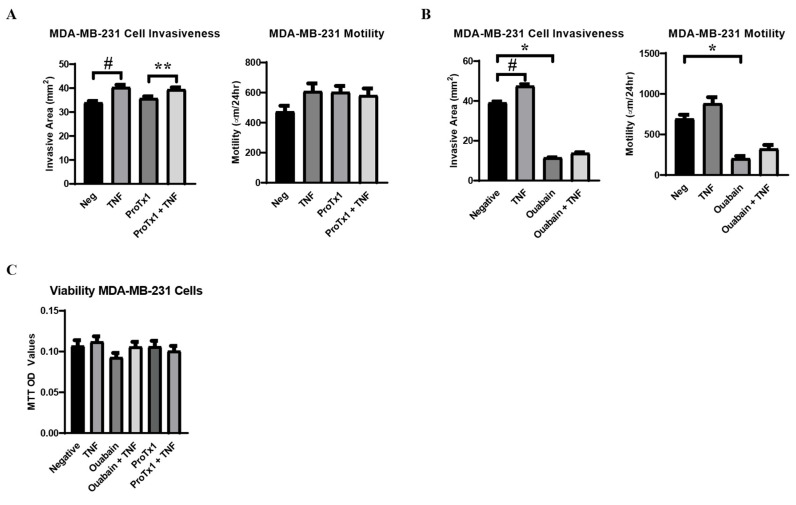
Metastasis-like behavior of MDA-MB-231 cells in response to TNFα, ProTx1, and ouabain. (**A**) Cell invasiveness (n = 18) and motility (n = 12) in cells treated with TNFα and ProTx1. (**B**) Cell invasiveness (n = 12) and motility (n = 12) in cells treated with TNFα and ouabain. Cell invasiveness was measured via the invasive area of MDA-MB-231 cells in a gel composed of agarose and supplemented cell culture medium (mm2), and cell motility measured via scratch test (µm/24 h) under control and experimental conditions. Significance determined via one-way ANOVA with a post-hoc Tukey’s multiple comparisons test (*, **, # *p* < 0.05). (**C**) Cell viability over time in response to incubation in control medium, or challenge with TNFα, ouabain, ouabain + TNFα, ProTx1, or ProTx1 + TNFα (n = 28). Significance between experimental groups and negative controls was determined via one-way ANOVA with a post-hoc Dunnett’s multiple comparisons test (*p* < 0.05).

Having demonstrated how TNFα, ProTx1, and ouabain modulated VGSC and Na,K-ATPase expression at the RNA and protein levels, the effect of these treatments on the metastatic-like behavior of MDA-MB-231 cells was examined. A scratch test was used to measure cell motility and an agarose-based assay was utilized to measure cell invasiveness. It was found that TNFα alone induced a significant increase in cell invasiveness and a trend towards increased cell motility, as was expected based on the previous literature (Figure 7A,B) [30,31]. ProTx1 induced no significant changes in cell motility and invasiveness in the cells (Figure 7A). Conversely, ouabain administration induced a significant decrease in both cell motility and invasiveness (Figure 7B). Co-administration of TNFα with ouabain induced trends towards increases in both cellular behaviors, but these effects were not significant and did not near the values of the negative controls. In order to determine whether these results were due to legitimate changes in cellular behavior or were simply artifacts of altered cellular proliferation, an MTT assay was used to measure cell viability over time in cells treated with TNFα, ProTx1, and ouabain. It was found that none of the experimental conditions significantly altered the viability of MDA-MB-231 cells over time, although there was a trend towards decreased viability in the ouabain treatment group (Figure 7C). In all, these data indicate that the treatments of TNFα, ProTx1, and ouabain can impact the in vitro metastasis-like behavior of MDA-MB-231 cells.

## 3. Discussion

Intracellular sodium concentration can have crucial impacts on cancerous growth and development in a variety of ways, from inducing cell proliferation, to promoting oncogenic gene expression, to triggering the efflux of H^+^ ions that degrade the extracellular matrix [2,3,4]. Voltage-gated sodium channels and Na,K-ATPase have both been implicated as metastatic drivers in a variety of cancers, but it is still largely unknown how they are regulated in cancer cells. Using the inflammatory regulation of ion transporters in pain-sensing neurons as a model, the present study examined whether these complementary proteins are co-regulated in metastatic breast cancer cells by inflammatory signals. We found that TNFα does indeed affect the regulation of VGSCs and Na,K-ATPase in MDA-MB-231 breast cancer cells at the levels of RNA expression, protein localization, and protein expression. Our findings provide evidence that these proteins are co-regulated in metastatic breast cancer in a mechanism that at least partly adheres to the proposed neuronal model. Inhibition of both the expression and ion-transporting function of Na_v_1.5 inhibits the acute inflammatory upregulation of sodium pump RNA, indicating that the inflammatory effect on the VGSCs leads to subsequent regulation of Na,K-ATPase. Similarly, the finding that sodium in serum-free Ringer’s solution is sufficient to recover the acuate inflammatory effect on Na_v_1.5 RNA but not Na,K-ATPase RNA, indicates that the inflammatory regulation of sodium pumps is downstream of sodium channels in breast cancer cells, as in neurons. Likewise, long-term ouabain administration decreased the protein expression of Na_v_1.5 in MDA-MB-231 cells just as it has been shown to decrease Na_v_1.8 expression in neurons [39]. Finally, chronic TNFα administration in MDA-MB-231 cells upregulates the overall expression of the Na,K-ATPase α3 subunit, which is normally expressed in neuronal cells. This is evidence that inflammatory signals may activate certain neural-specific genes in breast cancer cells, although this possibility requires further study. It should be noted that the magnitude of the inflammatory effect on Na_v_1.5 RNA was relatively modest, approximately a 20–60% increase above the untreated controls. This was observed consistently across multiple experiments, so the effect appears legitimate. However, in experimental conditions where TNFα had a trending but non-significant effect on Na_v_1.5 RNA, this might be either attributed to statistical noise in a particular experiment or a meaningful consequence of the experimental parameters. For example, while the siRNA knockdown of Na,K-ATPase α1 did not inhibit the inflammatory-mediated increase in Na_v_1.5 RNA, the effect of siRNA knockdown of the α3 subunit was less clear (Figure 1D). In these cases where the observed RNA effect is relatively consistent with previous experimental results that were found to be significant, the authors generally interpret these trending results as being indicative of a legitimate inflammatory-mediated increase in Na_v_1.5 RNA. However, further experiments will be necessary to confirm this.

In addition to the ways in which the inflammatory co-regulation of VGSCs and Na,K-ATPase in MDA-MB-231 cells adheres to our proposed neuronal model, we also found unexpected results that indicate a more complex regulatory mechanism. While acute TNFα administration increased RNA for Na_v_1.5, its overall protein expression was downregulated at all timepoints examined. In healthy depolarizing cells that express Na_v_1.5, such as cardiomyocytes, use-dependent internalization and proteasomal degradation are part of the protein’s regulatory cycle [38]. It may therefore be that any de novo production of Na_v_1.5 in MDA-MB-231 cells is outpaced by this internalization and degradation, as evidenced by the lower overall expression and increasingly diffuse staining pattern. The fact that ProTx1 administration, which blocks the influx of sodium ions through VGSCs, appears to inhibit the inflammatory-mediated downregulation of Na_v_1.5 at 1 h could support this theory, but more work is necessary to make a definitive conclusion. Conversely, ouabain administration potentiates the acute inflammatory effect on Na_v_1.5 in MDA-MB-231 cells at the RNA level and may contribute to an inflammatory increase in Na_v_1.5 protein at 1 h. These results, along with the observation that ouabain appears to inhibit the inflammatory-mediated upregulation of Na,K-ATPase α3 protein in these breast cancer cells, are evidence that the signal transduction functionality of sodium pumps can have both a positive feedback effect on VGSCs and an auto-regulatory effect in response to inflammatory stimulus. The observation that ProTx1 administration and siRNA knockdown of VGSCs raises basal levels of Na,K-ATPase RNA, while knockdown of Na,K-ATPase α1 and α3 raise basal levels of Na_v_1.5 RNA further demonstrates the complex co-regulation between these two ion transporters, which warrants further study.

Inflammation is strongly linked to cancer, as inflammatory conditions both acute, like viral infections, and chronic, like obesity, can contribute to the initiation or exacerbation of cancerous development [34,35]. The significant changes in the expression and localization of VGSCs and Na,K-ATPase demonstrated in these studies following TNFα administration are evidence that the regulation of sodium transporters may contribute to these inflammatory effects. Na_v_1.5 exhibited downregulated expression and diffusion of its localization in response to TNFα. Under control conditions, Na_v_1.5 consistently co-localized with the centrosome in MDA-MB-231 cells, but this was lost with chronic TNFα administration. The centrosomal localization of Na_v_1.5 may be related to the promotion of cell proliferation, or it may indicate a role of Na_v_1.5 in regulating cell migration, as has recently been demonstrated with the centrosomal localization of eIF2A in melanoma cells [40]. It is unclear whether the loss of centrosomal localization of Na_v_1.5 following TNFα challenge is due to an alteration of its cellular function or simply a consequence of its decreased overall expression, but the functions of Na_v_1.5 localization in MDA-MB-231 cells warrant further study. Interestingly there were divergent inflammatory effects on Na,K-ATPase subtypes. Na,K-ATPase α1, the most ubiquitous and robustly expressed subtype in human cells, did not change in its level of expression following TNFα administration. The α1 subunit appeared to increase its proportion of nuclear localization over time in culture in both the control and TNFα treatment groups, but this process appeared to be accelerated by inflammatory challenge in MDA-MB-231 cells. Expression of functional Na,K-ATPase within the nuclear envelope has been described in HEK293 cells, but it is unclear what is driving this altered localization in the MDA-MB-231 cells [41]. It could be that the nuclear localization of Na,K-ATPase α1 is associated with a particular phase of the cell cycle and that TNFα induces the cells to enter or remain in that phase. It may also be that the translocation of Na,K-ATPase α1 is part of a cellular response that is distinct from replication and is activated over base levels in response to inflammatory stimuli. Future experiments may be performed to explore these possibilities. Conversely, chronic inflammatory challenge did not alter the localization of Na,K-ATPase α3 but did increase the overall expression of that subunit. This is notable, not only because of the neural association of Na,K-ATPase α3, but because expression of α3 has previously been linked to survival and metastasis-like behavior in diverse cancer types [42,43,44]. It may be that the inducible upregulation of Na,K-ATPase α3 plays a significant role driving progression in a variety of cancers.

Administration of TNFα increased the metastasis-like behavior of the MDA-MB-231 cells, which is in line with the results of previous acute in vitro studies [30,31]. However, while previous studies documented decreased metastasis-like behavior in cancer cells treated with sodium channel-blocking drugs, we observed a non-significant trending increase in motility and invasiveness following ProTx1 administration [14,45]. These differences may be attributed to either the particular cell lines or the off-target effects of the drugs used in these studies. In any case, just as with the inconsistencies in the retrospective studies of human cancer patients on sodium transport-blocking drugs, these conflicting results underscore the importance of a more complete understanding of the regulation of sodium channels in metastatic cancer in order to target this system therapeutically [22,23,24,25]. Conversely, ouabain administration significantly decreased the metastasis-like behavior of the MDA-MB-231 cells, although it is unclear whether this effect was modulated by the ion transport-blocking or signal transduction effects of the drug on Na,K-ATPase. Regardless, the influence that Na,K-ATPase exerts over the aggressive behavior of these metastatic breast cancer cells along with the inflammatory co-regulation of sodium channels and sodium pumps, raises the possibility that the previously described effects of sodium channels on metastasis in cancer may not be due to direct VGSC activity but rather an indirect downstream effect on Na,K-ATPase. Sodium regulation remains a promising target to inhibit the progression of a wide variety of metastatic cancers, but a reductionist targeting of individual transporters seems insufficient to accurately describe this system. In order to realize the full potential of this therapeutic approach, more must be known about the integrated mechanisms regulating sodium transporters in cancer and the effects these proteins have on metastatic progression.

## 4. Materials and Methods

### 4.1. Cell Culture and Treatment

The human female metastatic breast cancer cell line MDA-MB-231 was acquired from ATCC (HTB-26). Cells were maintained in high glucose DMEM (Gibco, Waltham, MA, USA, 11995-065) supplemented with 10% fetal bovine serum (FBS) (Gibco, 26140-079) and 1% penicillin/streptomycin (pen/strep) (Gibco, 15070-063) at 5% CO2 and 37 °C. Cells were treated with 3.76 or 14.14 ng/mL TNFα (Gibco, PHC3016), 100 nM protoxin-1 (Alamone Labs, Jerusalem, Israel, STP-400), and 10 µM or 100 nM ouabain (Sigma-Aldrich, St. Louis, MO, USA, 03125). For Ringer’s experiments cells were washed in either Ringer’s buffer (Thermo Scientific, Waltham, MA, USA, AAJ67572AP) or sodium-free Ringer’s buffer (Thermo Scientific, AAJ67760AP), before being incubated in fresh Ringer’s buffer or sodium-free Ringer’s buffer for 30 min before adding experimental media. Transfection medium was prepared by combining siRNA against *SCN5A* (Santa Cruz, Dallas, TX, USA, SC-42640), *ATP1A1* (IDT, Coralville, IA, USA, 259214281), or *ATP1A3* (IDT, 259214675) with additive-free Opti-MEM (Gibco, 3185-070) and Lipofectamine 2000 transfection reagent (Invitrogen, Waltham, MA, USA, 11668-019). Regents were added to these solutions so that the transfected wells would receive 80 pmol/well of siRNA and 12.5 µL/well of Lipofectamine. Transfected cells were incubated for 24 h at 37 °C and incubated in normal supplemented culture medium overnight prior to 1 h inflammatory challenge.

### 4.2. RNA Extraction and qRT-PCR

For RNA extractions, cells were seeded at approximately 70% confluency and pre-incubated in 10µM insulin (Lonza, Basel, Switzerland, BE02-033E20) overnight prior to 1 h inflammatory challenge with 3.76 ng/mL TNFα. RNA was extracted with Quick-RNA MiniPrep kit (Zymo Research, Irvine, CA, USA, R1055). 25 ng of template RNA was used for each qRT-PCR reaction. Primers for the reference gene RER1 (F: 5′-CGTAGCGGAGCTGCGAG-3′; R: 5′-CGTGTAGGGTGTGGACTTGT-3′), *SCN5A* (F: 5′-CACGCGTTCACTTTCCTTC-3′; R: 5′-CATCAGCCAGCTTCTTCACA-3′), *ATP1A1* (F: 5′-TGTCCAGAATTGCAGGTCTTTG-3′; R: 5′-TGCCCGCTTAAGAATAGGTAGGT-3′) and *ATP1A3* (F: 5′-TCAGGACAACATCCCTGTGC-3′; R: 5′-GTATCGGTTGTCGTTGGGGT-3′) were added at a concentration of 200 nM per reaction, and all primer sequences were designed to span introns to ensure specific amplification of mRNA sequences without inadvertent genomic DNA amplification. Reactions were set up with an SYBR green one step qRT-PCR kit (Applied Biosystems, Waltham, MA, USA, 4389986) and run on a Lightcycler 480 II (Roche, Basel, Switzerland). Data from qRT-PCR is expressed either as a fold-change of a ΔCt or a ΔΔCt. Values for ΔCt were calculated for each sample by subtracting the reference gene Ct value from the target gene Ct value. For ΔCt expression, values were converted into a fold-change representing the RNA expression of the target gene relative to the RNA expression of the reference gene in each sample and was calculated as fold-change = 2^−ΔCt^. For ΔΔCt expression, values were calculated by subtracting the negative control ΔCt value from its paired experimental replicate ΔCt value (e.g., ΔCt TNFα Treatment Sample A − ΔCt Neg Control Sample A). The raw ΔΔCt values were converted into a fold-change of the experimental group target RNA expression relative to the negative control group target RNA expression as fold-change = 2^−ΔΔCt^. RNA expression compared to negative control RNA expression was assessed via one-tailed *t*-test (*p* < 0.05).

### 4.3. Immunocytofluorescence

MDA-MB-231 cells were seeded onto sterilized glass coverslips in a 6-well plate and treated with experimental medium (supplemented DMEM with or without 14.14 ng/mL TNFα). Plates were incubated at 37 °C for 1 h, 24 h, or 72 h. At each sample’s designated time-point, plates were soft-fixed with 100 µL of 1% paraformaldehyde solution (PFA) per 1 mL of cell culture medium in each well and incubated at 37 °C for 1 h. Plates were hard-fixed with 1% PFA for 10 min at room temperature before blocking (3% Bovine serum albumin, 5% goat serum) for 1 h at room temperature. Primary antibody-staining solutions were prepared in blocking buffer with overnight incubations at 4 °C. Primary antibodies were rabbit anti-Na_v_1.5 (Alamone Labs, ASC-005, 1:400), guinea pig anti-Na_v_1.5 (Alamone Labs, ASC-005-GP, 1:400), anti-Na,K-ATPase α1 (Alamone Labs, ANP-001, 1:200), anti-Na,K-ATPase α3 (Alamone Labs, ANP-003, 1:200), and anti-PCM-1 (Cell Signaling Technology, Danvers, MA, USA, 5213, 1:200). Secondary antibody Alexafluor 488 anti-rabbit (Invitrogen, A-11008, 1:1200) or Alexafluor 594 anti-guinea pig (Invitrogen, A-11076, 1:1200) diluted in blocking buffer was incubated for 45 min at room temperature. For anti-Na_v_1.5 samples, slides were counterstained with DRAQ5, mounted with prolong gold antifade reagent (Invitrogen, P36934), and images were taken on a Leica TS SP8 confocal microscope. For anti-Na,K-ATPase α1 and anti-Na,K-ATPase α3 samples, slides were mounted with Prolong Gold antifade reagent with DAPI as a nuclear counterstain (Invitrogen, P36935), and images were taken on a Nikon Eclipse Ti2 fluorescent microscope. Cells stained for Na,K-ATPase α1 were manually counted to quantify cells exhibiting nuclear co-localization. For each image taken, the percentage of cells displaying nuclear localization of Na,K-ATPase α1 was graphed relative to the total number of cells in the image. This was performed for 1 h Neg (n = 6 images), 1 h TNF (n = 4 images), 24 h neg (n = 4 images), 24 h TNF (n = 5 images), 48 h neg (n = 7 images), and 48 h TNF (n = 7 images). Statistical significance was assessed via two-tailed *t*-test (*p* < 0.05).

### 4.4. Western Blot

MDA-MB-231 cells were seeded into t75 tissue culture flasks. Cells were treated in experimental media and incubated at 37 °C before protein was extracted at 1 h, 24 h, and 48 h. Experimental media were comprised of supplemented DMEM with added 14.14 ng/mL TNFα, 100 nM ProTx1, and 100 nM ouabain (Ouabain). Protein was extracted with Transmembrane Protein Extraction Reagent (TPER) (FIVEphoton, San Diego, CA, USA, TmPER-200) supplemented with cOmplete mini protease inhibitor tablets (Roche, 11836153001). For Na_v_1.5 detection, 35 µg/well was loaded into a 1.5 mm NuPAGE Tris-acetate gradient gel (Invitrogen, EA038BOX) before wet-transfer was performed in Tris-glycine transfer buffer (25 mM Tris-base, 192 mM Glycine, 0.05% SDS, 5% MeOH, pH 8.3) run at 0.3A under refrigerated conditions with PVDF membranes that had been pre-wetted in methanol. Membranes were blocked in 5% Non-fat Dry Milk TBS and stained with rabbit anti-Na_v_1.5 antibody (Cell Signaling Technologies, 14421, 1:200) and rabbit anti-GAPDH (Invitrogen, PIPA585074, 1:1000) antibodies. For Na,K-ATPase α1 and Na,K-ATPase α3 detection, 8.75 µg/well and 17.5 µg/well, respectively, were loaded into 1 mm Mini-PROTEAN TGX stain-free gradient gels (Bio-rad, Hercules, CA, USA, 4568093) and transferred with a Transblot Turbo PVDF transfer pack (Bio-rad, 1704156). Membranes were blocked and stained with a rabbit anti-Na,K-ATPase α1 antibody (Cell Signaling Technologies, 23565, 1:500), or mouse anti-Na,K-ATPase α3 antibody (Thermo Fisher, Waltham, MA, USA, MA3-915, 1:8000). Secondary antibody incubations were performed for 1 h at room temperature at a 1:2500 dilution (Bio-rad, StarBright Blue 700 goat anti-rabbit, 12,004,162 and StarBright Blue 700 goat anti-mouse, 12004159). Blots were imaged on a ChemiDoc MP system (Bio-rad) and analyzed on Image Lab software (Bio-rad, Version 6.1.0 build 7). The volume of the Na_v_1.5 band detected for each sample was normalized to the volume of the GAPDH band from the same sample. For Na,K-ATPase α1 and α3, total protein was detected on the PVDF membrane following transfer utilizing the stain-free total protein detection of the Bio-rad TGX gels. The band volume for each target protein was then normalized to the total protein in each lane. Data is presented as a fold-change of normalized band volume of the treatment groups relative to the normalized band volume of a paired negative control. Statistical significance between every experimental group relative to negative controls was assessed via one-way ANOVA with post-hoc Dunnett’s multiple comparisons tests (*p* < 0.05). Individual treatment groups were also graphed with the normalized band volumes of each TNFα-treatment group relative to its paired non-TNFα-treated controls. Statistical significance was assessed via two-tailed *t*-tests (*p* < 0.05).

### 4.5. Motility and Invasiveness

Analysis of cell motility of MDA-MB-231 cells under experimental conditions was assessed via scratch test [46]. Cells were seeded into 6-well plates at a concentration of 1 × 106 cells/well. Cells were incubated in experimental media overnight at 37 °C. Experimental media conditions were supplemented DMEM with added 14.14 ng/mL TNFα, 100 nM ProTx1, or 100 nM ouabain. Cell monolayers were scratched with a p200 pipette tip, at which point plates were washed gently in PBS and seeded with fresh experimental media. Scratches were imaged with a Moticam 5.0 MP microscope camera under 100× total magnification at t = 0 h and t = 24 h, then analyzed using Motic Images Plus 3.0 software (Motic, Hong Kong, China). For each image, the distance between the cell lines was measured in triplicate (µm), and these values were averaged. The average distance between the cell lines at t = 24 was subtracted from the average distance between the cell lines at t = 0 to calculate the cell motility (µm/24 h) for each sample. Mean cell motility values were calculated for each experimental group (n = 12), and significance in motility values between experimental groups was calculated with a one-way ANOVA with a post-hoc Tukey’s multiple comparisons test (*p* < 0.05).

Cell invasiveness in MDA-MB-231 cells under experimental conditions was assessed via an agarose invasive area assay, as previously described [47]. In brief, a 24-well plate was seeded with a gel solution made of a 1:1 ratio of 1% agarose in PBS and supplemented cell culture medium. The agarose gel was made either with supplemented DMEM (neg) or with additives to reach a final concentration of 14.14 ng/mL TNFα, 100 nM ProTx1, 100 nM ProTx1 with 14.14 ng/mL TNFα, 100 nM ouabain, or 100 nM ouabain with 14.14 ng/mL TNFα. After the gel solution solidified in the well-plate, a hole was punched in the gel using a cutoff 1 mL serological pipette. Cells were harvested, pelleted, and resuspended in serum-free DMEM. Cell suspensions were then counted, and 4 × 10^4^ cells were seeded into the punched hole in the gel of each well. The plates were incubated at 37 °C for seven days, during which time the cells invaded into the semi-solid matrix of the agarose gel. At d7, the wells were fixed in 10% neutral buffered formalin, washed in PBS, and stained with cresyl violet solution. Using a light microscope at 40× total magnification, the distance the cells invaded into the agarose was marked at four cardinal points on the bottom of the well plate. The distance between two of these marks was measured (mm) with an electronic caliper, the plate was rotated 45° and the distance between the second two marks was measured. These two distances represent the diameters of the invasive area of the cells, and the invasive area of the cells was calculated as area = (diameter1 ÷ 2) × (diameter2 ÷ 2) × π. The mean invasive areas for each experimental group were calculated and the significance of the difference between experimental groups (n = 12 for ouabain experiments, n = 18 for ProTx1 experiments) was assessed with a one-way ANOVA with a post-hoc Tukey’s multiple comparisons test (*p* < 0.05).

### 4.6. Cell Viability

The proliferation and survival of MDA-MB-231 cells over time in experimental media was measured with a CYQUANT MTT assay (Invitrogen, V13154). Cells were seeded in a 96-well plate at 5000 cells/well. Cells were then challenged with experimental media and incubated overnight at 37 °C. Experimental media consisted of supplemented DMEM with no 14.14 ng/mL TNFα, 100 nM ProTx1, or 100 nM ouabain. Cells were incubated in experimental media made with 1× PBS instead of DMEM with the same additives in order to prevent assay interference from phenol red. MTT solution was then added to the wells and the plate was incubated overnight at 37 °C. The following day, an SDS-HCl solution was mixed in each well and the plate was incubated for 4 h at 37 °C at which point the OD values in the wells were read at 570 nm. An average value was calculated from 4 blank control wells (PBS + MTT solution + SDS-HCl solution with no cells), and this value was subtracted from the OD value of each experimental well (n = 8 for each condition). These values were averaged for each experimental group and statistical significance between each experimental group and the negative controls was calculated via one-way ANOVA with a post-hoc Dunnett’s multiple comparisons test (*p* < 0.05).

### 4.7. Statistical Analyses

All statistics were performed and graphs were generated in GraphPad Prism 10 (GraphPad Software, LLC, Boston, MA, USA, Version 10.2.3).

## Data Availability

The raw data supporting the conclusions of this article will be made available by the authors on request.

## Abbreviations

The following abbreviations are used in this manuscript:

VGSC	Voltage-gated sodium channels
Na,K-ATPase	Sodium-potassium ATPase
TNFα	Tumor necrosis factor alpha
ProTx1	Protoxin-1
ERK1/2	Extracellular signal-regulated kinase 1/2
EGFr	Epidermal Growth Factor Receptor
